# Soot Combustion over Nanostructured Ceria with Different Morphologies

**DOI:** 10.1038/srep29062

**Published:** 2016-06-29

**Authors:** Wen Zhang, Xiaoyu Niu, Liqiang Chen, Fulong Yuan, Yujun Zhu

**Affiliations:** 1 Key Laboratory of Functional Inorganic Material Chemistry (Heilongjiang University), Ministry of Education, School of Chemistry and Materials, Heilongjiang University, Harbin, 150080 P. R. China

## Abstract

In this study, nano-structure ceria with three different morphologies (nanorod, nanoparticle and flake) have been prepared by hydrothermal and solvothermal methods. The ceria samples were deeply characterized by XRD, SEM, TEM, H_2_-TPR, XPS and *in-situ* DRIFTS, and tested for soot combustion in absence/presence NO atmospheres under loose and tight contact conditions. The prepared ceria samples exhibit excellent catalytic activities, especially, the CeO_2_ with nanorod (Ce-R) shows the best catalytic activity, for which the peak temperature of soot combustion (T_m_) is about 500 and 368 °C in loose and tight contact conditions, respectively. The catalytic activity for Ce-R is higher than that of the reported CeO_2_ catalysts and reaches a level that of precious metals. The characterization results reveal that the maximal amounts of adsorbed oxygen species on the surface of the nanostructure Ce-R catalyst should be the crucial role to decide the catalytic soot performance. High BET surface area may also be a positive effect on soot oxidation activity under loose contact conditions.

Diesel engines are of great significance to modern society due to their optimal fuel efficiency, low emission of CO_2_ and high durability. They are widely used to vehicles, ships and working machines, however, emission of particulate matter (PM) and nitrogen oxides (NO_x_) in the process of combustion has been received attention due to causing massive problems to the environment and health, one of them is carbonaceous soot as a major pollutant^1^,^2^,^3^. Therefore, that calls for efficient exhaust gas treatment systems to reduce soot.

The catalytic soot oxidation is one of the most efficient techniques to reduce emission of soot particles. Several kinds of catalysts have exhibited good catalytic performances for diesel soot combustion, such as mixed metal oxides combined with noble metals^3^,^4^,^5^,^6^, alkaline metal oxides^7^,^8^, transition metal oxides^9^,^10^,^11^, perovskite-like type oxides^12^,^13^,^14^ and ceria-based oxides^1^,^15^,^16^,^17^,^18^,^19^,^20^,^21^,^22^,^23^. Among these catalysts, precious metals have good catalytic activity, but they are most expensive. Zhao *et al*. have carried out many valuable researches on the removal of soot^4^,^6^,^21^,^23^,^24^,^25^. A series of three dimensionally ordered macroporous (3DOM) catalysts (Pt/TMO, 3DOM Pt/Fe_2_O_3_ and Pt/Co_3_O_4_) were prepared and exhibited much higher catalytic activity for diesel soot combustion in contrast to other catalysts. In addition, CeO_2_ has the excellent redox properties, oxygen storages and release capability by the way of facile cycle conversion between Ce^3+^ and Ce^4+^ oxidation states^1^,^26^,^27^,^28^,^29^. Particularly, inserting transition metals into a CeO_2_ framework can increase CeO_2_ thermal stability and the number of defects which improve its redox properties. CeO_2_ doped with transition metals is considered as a good cheap substitute for noble metals catalyst for soot oxidation.

In recent years, numerous studies have shown that CeO_2_ with shapes has a positively effect on catalytic performances such as photocatalytic and hydrogen electro-oxidation^30^,^31^,^32^. In this context, CeO_2_ could also act as a catalyst in soot oxidation reactions and its physicochemical properties can be influenced by their structural properties^32^,^33^. Therefore, it is meaningful to prepare CeO_2_ catalysts with different shapes and morphologies for investigating their catalytic performances of soot combustion because the activity can be correlated to the exposure of reactive crystal planes and active species in different shapes CeO_2_ nanoparticles^32^,^34^,^35^,^36^,^37^,^38^,^39^,^40^. Recently, Trovarelli *et al*.^37^ synthesized two types of shape-controlled CeO_2_ (nanocubes and nanorods) and compared their catalytic performance for soot combustion. In addition, different morphologies influenced the structure defects of ceria. Bensaid *et al*.^38^ reported a hydrothermal method to synthesize CeO_2_ with self-assembled stars morphologies, which had high specific surface area (105 m^2^/g) and improved the soot-catalyst contact to enhance the activity of soot combustion. Thus, it is meaningful to prepare different morphology of CeO_2_ in order to improve its catalytic performance by controlling its surface structure.

Based the effect of shapes on surface physicochemical properties, our present study aims are preparation of CeO_2_ with different morphologies and investigation on the relation between the catalytic performance for soot combustion and the shapes. In this work, CeO_2_ with three kinds of morphologies including nanorod, nanoparticle and nanoflake were synthesized and justified their catalytic behavior for soot oxidation under different contact conditions in absence/presence NO atmospheres. The CeO_2_ with nanorod exhibited the best catalytic activity, and it reached a level that of a precious metal catalyst.

## Results

### Textural properties

**Figure 1:**
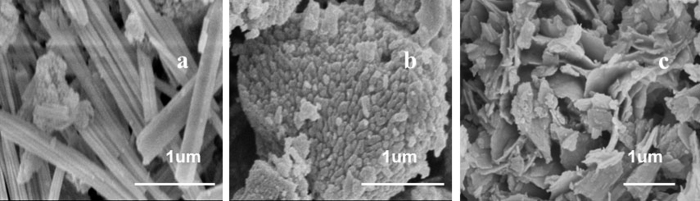
SEM images of (**a**) Ce-R, (**b**) Ce-P and (**c**) Ce-F.

The morphologies of as-prepared CeO_2_ with different preparation methods were investigated by the SEM shown in Fig. 1. The morphologies of the CeO_2_ catalysts exhibit an outstanding reliance on the preparation methods. SEM images display three different morphologies of CeO_2_: nanorods (Ce-R), nanoparticle (Ce-P) and flakes (Ce-F). They were successfully prepared by hydrothermal method and solvothermal method. Figure 1a shows the nanorods with a hierarchical structure consist of many small bars with various thicknesses and lengths. The small bars have uniform diameters of 20–35 nm and form a rod with width of 250 nm and with length of about 2 μm. So much lengths of nanorods were even longer than that of others reported in literatures (300–350 nm)^37^,^39^. For the Ce-P, the small nanoparticles are about 30–40 nm in diameter and tightly together to form a spherical shape exhibited in Fig. 1b. Figure 1c displays the flower consists of flakes for the Ce-F and the flakes with each petal thickness are approximate 25 nm.

**Figure 2:**
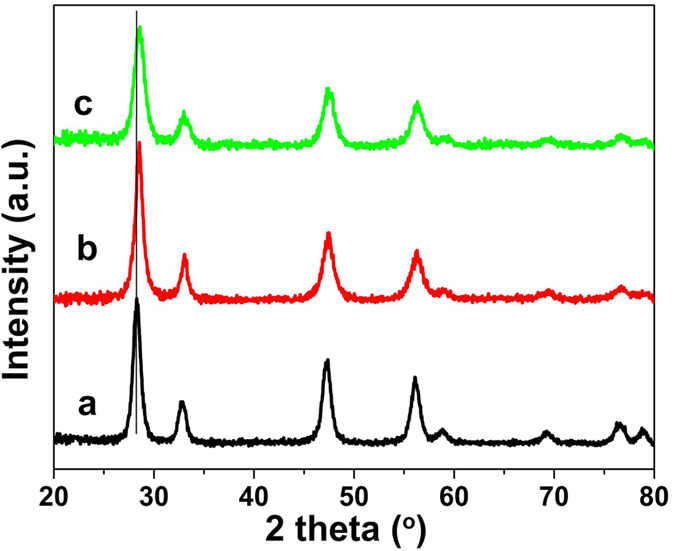
XRD patterns of (**a**) Ce-R, (**b**) Ce-P and (**c**) Ce-F.

Figure 2 reports the XRD patterns of the Ce-R, Ce-P and Ce-F samples. Their patterns are very similar and the diffraction peaks located at 28.4, 32.8, 47.4, 56.3, 58.8, 69.3, 76.5 and 79.3^o^ that can be assigned to the ceria fluorite structure of the (111), (200), (220), (311), (222), (400), (331) and (420) crystalline faces (JCPDS 65-5923), respectively. Compared to the Ce-P and Ce-F samples, the diffraction peaks of the Ce-R shifted to a low *2θ* angle, indicating the increase in lattice parameters. Rietveld refinements analysis of the XRD profile was carried out (Fig. S1). The lattice parameter was calculated and the obtained results were presented in Table 1. The calculated lattice parameter is approximately 5.4375, 5.4164 and 5.4227 Å for Ce-R, Ce-P and Ce-F, respectively, which are bigger than that of reported for conventional ceria (5.4110 Å)^37^, suggesting the lattice expansion for Ce-R, Ce-P and Ce-F. This is most likely due to the existence of more Ce^3+^ in the crystal lattice because the radius of Ce^3+^ cations (1.14 Å) is larger than that of Ce^4+^ (0.97 Å) inducing to the lattice expansion^41^,^42^,^43^. According to the XRD results, Ce-R with nanorod structure may possess more defective structures among these CeO_2_ samples due to the existence of more Ce^3+^ amounts.

**Figure 3:**
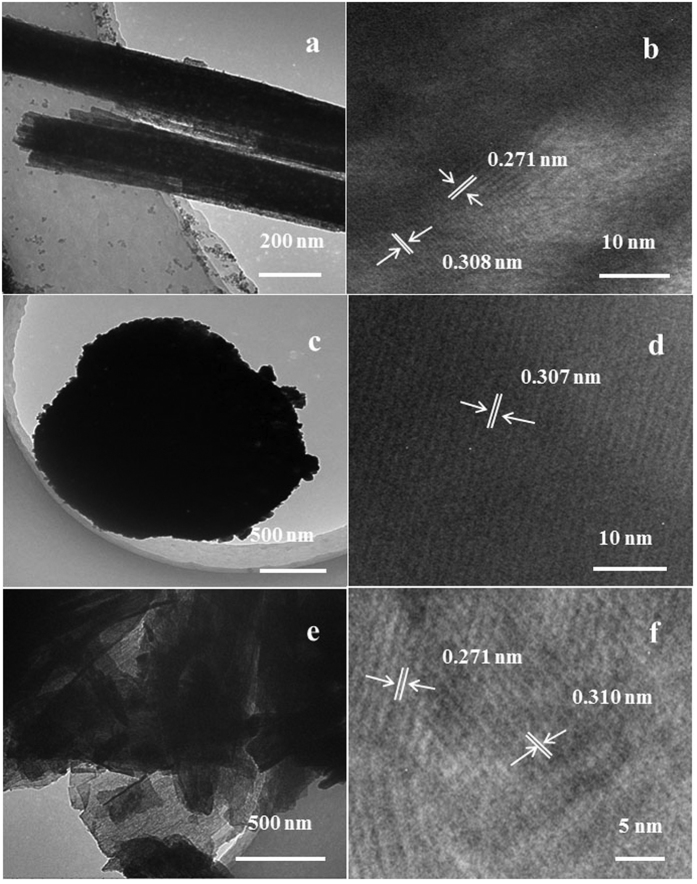
TEM images of (**a,b**) Ce-R, (**c,d**) Ce-P and (**e,f**) Ce-F.

Furthermore, these typical samples were also carefully studied by TEM. The TEM and HRTEM images of the Ce-R sample are reported in Fig. 3a,b. It can be clearly seen that a separate rod composed by small bars from TEM image of Ce-R (Fig. 3a), whereas the HRTEM image (Fig. 3b) shows the clear lattice fringes with inter-planar spacing of 0.308 and 0.271 nm which are assigned to the (111) and (100) crystal planes of CeO_2_, respectively^36^,^37^,^44^. In Fig. 3c, Ce-P shows a spherical nano-structure, which is accumulated by amount of small nanoparticles. The diameter of spherical particles is about 2 μm, which is consistent with the SEM results (Fig. 1b). After carefully calculation, it is deduced that the dominant lattice fringes spacing of 0.307 nm correspond to the (111) crystal planes of CeO_2_^36^,^37^ judging from the representative HRTEM image in Fig. 3d. In the TEM image of the Ce-F sample (Fig. 3e), it is clearly observed that the Ce-F possesses flaky structures. The lattice fringes spacing is also calculated to 0.271 and 0.310 nm in the Fig. 3f, which should be recognized as the (100) and (111) crystal plane of CeO_2_, respectively^36^,^37^,^44^., In addition, other region of TEM images for Ce-R and Ce-F are shown in Fig. S2. Combined with XRD results, it confirmed the polycrystalline structure for these CeO_2_ samples. The three kinds of samples are not optimal oriented growth along with a crystal plane to form single crystalline. The differences in morphologies of as-prepared CeO_2_ are due to nanoparticles accumulation in a different way with different preparation methods.

### H_2_-TPR characterization

**Figure 4:**
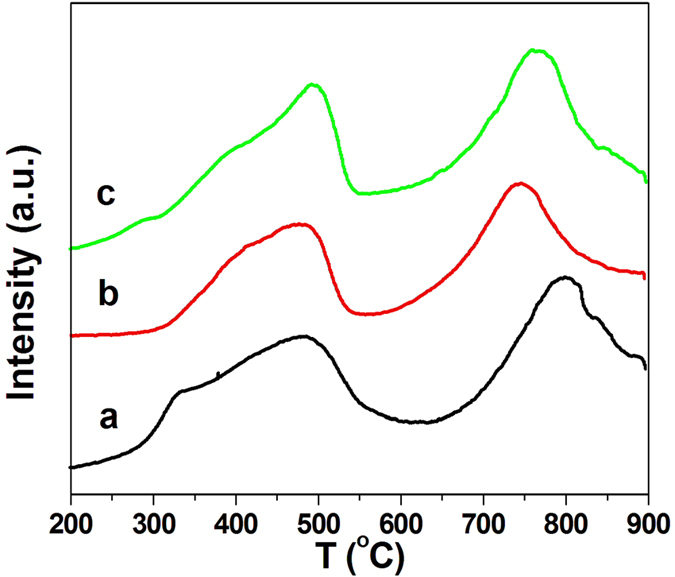
H_2_-TPR curves of (**a**) Ce-R, (**b**) Ce-P and (**c**) Ce-F.

The reducibility of Ce-R, Ce-P and Ce-F was studied by H_2_-TPR shown in Fig. 4. The H_2_-TPR results of all samples exhibit a bimodal shape with a wide low temperature peak (T_1_) at 300–600 °C and a wide high temperature peak (T_2_) at 700–900 °C, which is attributed to the characteristic reduction of surface oxygen and bulk oxygen reduction, respectively^38^,^39^,^45^. Overall reduction degree, the hydrogen consumption was calculated at different temperature range by integrating H_2_-TPR profiles listed in Table 1. The H_2_ uptake for the low-temperature reduction peak increases with the order of Ce-R (0.72 mmol·g_cat_^−1^) > Ce-F (0.61 mmol·g_cat_^−1^) > Ce-P (0.48 mmol·g_cat_^−1^). It can be seen that the Ce-R possess the largest amount of surface oxygen species among these samples. There is a difference between the top temperatures of the prepared ceria, however, the high-temperature peak is broad and the hydrogen consumption has relatively small deviations in range of 0.39–0.45 mmol·g_cat_^−1^. Therefore, the prepared CeO_2_ sample exhibits similar reactivity of lattice oxygen.

In addition, O_2_-TPD measurements were carried out and the results are shown in Fig. S3. For all the prepared ceria samples, a large and wide peak at the temperature range of 100–600 °C was observed, which was assigned to a continuous desorption of the surface oxygen. The area of peaks by integrating the O_2_-TPD profiles was 1159, 805 and 774 for Ce-R, Ce-P and Ce-F, respectively, indicating that there is more surface oxygen species on the surface of Ce-R.

### XPS characterization

**Figure 5:**
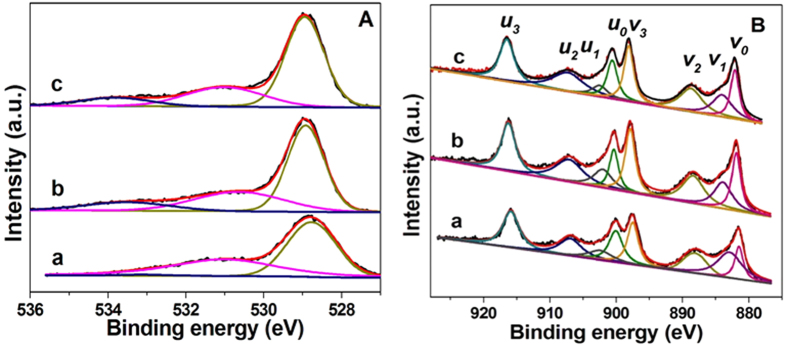
XPS of O 1s (**A**) and Ce 3d (**B**) for (a) Ce-R, (b) Ce-P and (c) Ce-F.

XPS was used to investigate the surface chemical properties of all ceria samples in terms of oxidation states and species of surface atoms. The O1s spectra are shown in Fig. 5A, together with their deconvolution obtained by fitting Gaussian peaks after Shirley-background subtraction. The O1s spectra show different features, which depends on both the chemisorbed oxygen species (O_α_) and lattice oxygen (O_β_). Here, two kinds of surface oxygen species (O_α_) were identified. The binding energy at 530.5–531.4 eV can be attributed to the defect oxide or the surface oxygen species (O_α1_) adsorbed on the oxygen vacancies (i.e. O_2_^2−^, O^−^, OH^−^, CO_3_^2−^), and the binding energy at 532.7–533.8 eV is assigned to adsorbed oxygen species from hydroxyl species and adsorbed water species (O_α2_) on the surface^46^. The binding energy at 528.8–529.1 eV is ascribed to lattice oxygen (O_β_). In addition, in terms of the quantitation of the surface oxygen species, the ratio of O_α1_ to O_β_ (O_α1_/O_β_) was calculated according to the peak area shown in Table 1. The results present the decrease in the O_α1_/O_β_ value according to the order of Ce-R (0.87) > Ce-F (0.77) > Ce-P (0.74), which is almost consistent with the order of hydrogen consumption at low temperature in H_2_-TPR (Supplementary Fig. S4). It is confirmed that the surface adsorbed oxygen species of the Ce-R sample is more than that of the others.

Figure 5B shows the XPS spectra in the Ce3d region that was divided into 8 peaks by peak-fitting deconvolution for each sample. The 3d_5/2_ is corresponding to *v*, while the 3d_3/2_ is corresponding to *u*. The doublets (*v*_*0*_, *u*_*0*_), (*v*_*2*_, *u*_*2*_), and (*v*_*3*_, *u*_*3*_) represent 3d^10^4f^0^ initial electronic state that are assigned to Ce^4+^, while the signals (*v*_*1*_, *u*_*1*_) represent 3d^10^4f^1^ state of Ce^3+ ^39,47. Therefore, the relative abundance of the Ce^3+^ (%) species of each sample has been estimated by considering the deconvolution peaks of Ce3d binding energies. In Table 1, Ce^3+^ content is calculated as 25.1%, 19.1% and 16.5% for the Ce-R, Ce-F and Ce-P, respectively. The order follows as: Ce-R > Ce-F > Ce-P, which indicating that the largest Ce^3+^ content (namely 25.1%) on the Ce-R surface among these samples. It is reported that the existence of Ce^3+^ in CeO_2_ is associate with the formation of oxygen vacancies^39^,^46^. The larger Ce^3+^ content are observed, the more oxygen vacancies are formed on the surface of the prepared CeO_2_ samples. Therefore, the Ce-R sample has the largest Ce^3+^ content on its surface, which implies a relatively higher amount of structure-defects on the surface of the Ce-R. It is favor of formation of surface adsorbed oxygen species. This result is also consisting with the O1s of XPS, H_2_-TPR and O_2_-TPD results.

### *In situ* DRIFTS characterization

**Figure 6:**
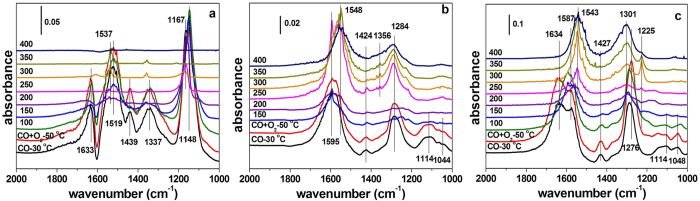
*In situ* DRIFTS of (**a**) Ce-R, (**b**) Ce-P and (**c**) Ce-F (a flow of 0.5vol%CO/N_2_ before and after the addition of 3% O_2_/N_2_ at different temperature).

In order to investigate the reactivity of the surface species of the prepared CeO_2_ sample, *in situ* DRIFTS were carried out over these ceria samples under a flow of 0.5vol%CO/N_2_ and 3vol%O_2_/N_2_ at different temperatures (Fig. 6).

Figure 6a shows *in situ* DRIFTS of Ce-R. When Ce-R sample adsorbed CO at 30 °C, some characteristic peaks ascribed to the carbonate species appeared at the bands of 1633, 1537, 1519, 1439, 1337, and 1148 cm^−1^. The bands at 1633 and 1537 cm^−1^ are assigned to bidentate carbonates, while the bands at 1439, 1337 and 1148 cm^−1^ correspond to monodentate carbonates^48^,^49^,^50^. The peak at 1519 cm^−1^ is related to inorganic carboxylate^48^,^49^,^50^,^51^. Adding 3vol%O_2_/N_2_ into the feed, the characteristic peaks at 100 °C were in accordance with that of adsorbed CO at 30 °C. When the Ce-R sample was heated from 100 °C to high temperature, the intensities of these peaks began to decrease and gradually disappeared above 300 °C, which suggests that carbonate species on the Ce-R surface occurs to desorption or decomposition.

Figure 6b displays *in situ* DRIFTS of Ce-P. The IR features observed at the beginning of CO adsorption at 30 °C can be assigned to a mixture of bidentate (1595 and 1284 cm^−1^), unidentate (1114 and 1044 cm^−1^) carbonate species^48^. The peak at 1424 cm^−1^ is related to inorganic carboxylate^48^,^49^,^50^,^51^. The spectra displayed sensitive changes in intensity as a function of temperature. After the addition of 3vol%O_2_/N_2_ at 50 °C, the spectra appeared to be very similar to the CO adsorption at 30 °C. With increasing the temperature from 150 °C to 250 °C, the sharp peak at 1595 cm^−1^ became stronger and stronger. At temperature of 250 °C and above, new carbonate-type species at 1548 and 1356 cm^−1^ were observed, which were assigned to bidentate carbonates and monodentate carbonates, respectively^48^. It may be CO reacted with lattice oxygen of ceria even in the presence of gaseous O_2_ to form new carbonates^52^. When the temperature got to 400 °C, there were also some residual carbonates-like species on the surface of Ce-P, indicating the species were not easy to desorb or decompose.

Figure 6c shows DRIFTS of the Ce-F sample. It appeared similar peaks and evolution of the carbonate species to the Ce-P sample with the reaction temperature. After adding into 3vol%O_2_/N_2_ at 50 °C, the characteristic peaks were in the same with that of adsorbed CO at 30 °C, however, the former was stronger than the latter. The weak peaks assigned to monodentate carbonates at 1114 and 1048 cm^−1^ and inorganic carboxylate at 1427 cm^−1^ became vanish at 150 °C. Specifically, gradual redshift of these peaks was observed with the increase in the temperature and following bands ascribed to bidentate carbonates at 1643–1587 and 1587–1543 cm^−1^, at the same time, the monodentate carbonates band at 1276 cm^−1^ almost removed to high wavenumber a little and can be observed two new bands at 1301 and 1225 cm^−1^. It may be carbonates decomposition and CO interacting with surface oxygen/lattice oxygen form new carbonates simultaneously with the reaction of temperature^52^. With further increasing the temperature, the peaks intensities of carbonate species increase and the residual carbonate species on the surface of Ce-F catalyst also can be seen.

In the DRIFTS (Fig. 6), it observed that carbonates were identified on the samples, but not bicarbonates, although a little hydroxyl content exist the samples surface was suggested in the XPS. It may be the hydroxyl were purge away when the pretreated with N_2_ stream.

### Soot oxidation activity

**Figure 7:**
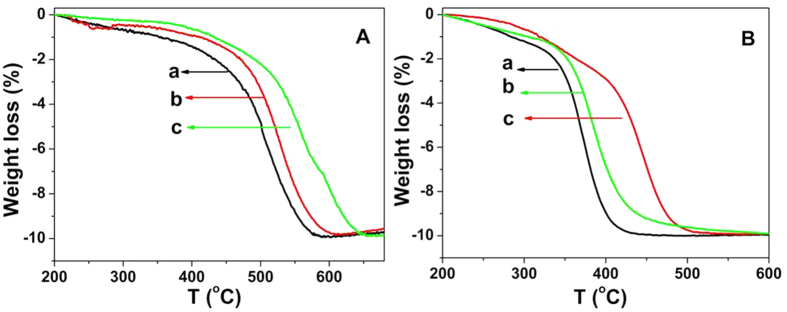
Weight-loss profiles of soot over (a) Ce-R, (b) Ce-P and (c) Ce-F (**A**) under loose contact and (**B**) tight contact in 10vol%O_2_/N_2_.

Soot combustion reaction is a typical heterogeneous catalytic reaction containing solid particle as reactant. The catalytic activity is related to the temperature at which 10%, 50% and 90% of weight loss is observed (denoted as T_10_, T_50_ and T_90_, respectively). The weight-loss curves showing the decomposition profile of the soot mixed with the Ce-R, Ce-P and Ce-F under the loose contact and tight contact are shown in Fig. 7A,B, respectively. The T_10_, T_50_ and T_90_ values of the catalysts are reported in Table 2. The T_10_, T_50_ and T_90_ values of Ce-R catalyst are 356, 500 and 554 °C, which are lower than that of Ce-P and Ce-F (Table 2 and Fig. 7A), indicating Ce-R exhibits much higher activity for soot combustion under loose contact condition. The catalytic activity improves under loose contact condition with the order of Ce-R > Ce-P > Ce-F.

Generally, the mechanical force generates a particularly close contact between the soot and the catalyst in tight contact. Figure 7B illustrates the weight-loss profiles of soot combustion under the tight contact between soot and catalyst. The Ce-R catalyst exhibits the highest catalytic activity for soot oxidation among these catalysts, in which the lowest T_10_, T_50_ and T_90_ are 286, 368 and 400 °C, respectively (Table 2). Additionally, the catalytic performances of Ce-R were compared with some CeO_2_ catalysts that reported in the literatures^37^,^38^,^40^,^53^ which are listed in Supplementary Table S1. In our work, the T_m_ (temperature at the maximal soot combustion rate) values are 372, 439 and 383 °C for Ce-R, Ce-P and Ce-F, respectively. It is found that the T_m_ values in the literatures are above 400 °C, especially, the T_m_ values of CeO_2_ with nanorod structure reported by Trovarelli *et al*. is 405 °C^38^, but it is 368 °C for the Ce-R catalyst. It is clearly seen that the Ce-R possesses an obvious advantage with a much lower T_m_ value evaluated under the tight contact conditions.

**Figure 8:**
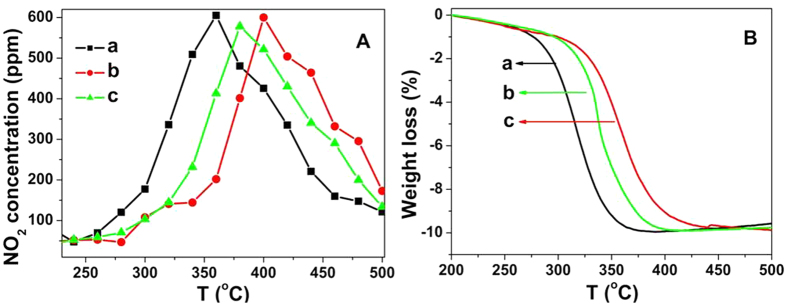
Evolutions of NO_2_ concentration (**A**) and the TGA profile (**B**) for (a) Ce-R, (b) Ce-P and (c) Ce-F mixed with soot under the tight contact condition in 1000 ppm NO and 10vol%O_2_/N_2_.

Diesel exhaust contains generally both soot and NO_x_ as two major pollutants. Thus, the soot combustion with the addition of NO_x_ was also tested under the tight contact over the above three catalysts. The results of NO oxidation are shown in Fig. 8A and Table 3. The temperature at which the maximal NO_2_ concentration attained in the outlet gas was denoted as T_m_(NO_2_). The T_m_(NO_2_) of Ce-R, Ce-P and Ce-F are 360, 399 and 379 °C, respectively. These results indicate the NO oxidation is remarkable over the Ce-R catalyst.

The weight-loss curves of soot mixed with CeO_2_ catalysts with the different morphologies are shown in Fig. 8B and Table 3 under tight contact condition in the presence of NO and O_2_. The T_10_ of Ce-R, Ce-P and Ce-F are 275, 301 and 296 °C, respectively. The weight losses of soot combustion for the three catalysts in the presence of NO follow the same order of in the absence of NO. But the soot combustion activities are superior to that in absence of NO conditions. Compared Fig. 8A with Fig. 8B, NO_2_ concentration reaches the maximum value when the soot is burned completely. Because NO_2_ is a more powerful oxidant than O_2_ for soot oxidation^18^,^19^, leading to the NO conversion to NO_2_ efficiently decrease the soot combustion temperature. It implies the consumption of NO_2_ by reaction with soot at low temperatures which form the surface oxygenated species C(O). Afterwards, the C(O) can either be decomposed to release CO or be oxidized by O_2_ to produce CO_2_. When the soot reaction is finished, the main production is NO_2_.

The above results suggest that the prepared ceria with different morphology exhibits different activity for soot combustion under either loose or tight contact condition. The CeO_2_ nanorods exhibit excellent catalytic activity for soot combustion.

## Discussion

For catalytic soot oxidation, some authors attribute a key role to the surface/textural properties of ceria^39^,^42^,^43^, and others agree in explaining the activity of cerium oxide with its oxygen storage and redox capacity^2^,^38^,^54^,^55^. One of the most important roles of CeO_2_ in catalytic redox reaction is to provide surface active sites and to act as an oxygen buffer providing oxygen storage/transport by shifting between Ce^4+^ and Ce^3+^ during reaction. In addition, many researchers reported that the contact conditions between the catalyst and soot particles also affect the catalytic performance of soot oxidation^25^,^38^,^53^.

In this work, we have successfully prepared three different morphologies ceria catalysts (nanorods, nanoparticles and flakes). The soot combustion has been carried out under tight and loose contact conditions between soot and catalyst. The experiment results show that catalytic performance of soot oxidation in tight contact condition is superior to that in loose contact. It is consistent with the results reported by many researchers^38^,^39^,^40^,^53^. The catalytic activities of as-prepared ceria are much better than that of most of ceria reported in the literatures^37^,^38^,^40^,^53^. We suggest that their redox capacity, the amount of surface oxygen species and the surface area etc. have positively correlated relations with soot oxidation activity according to the above characterizations. From H_2_-TPR results (Fig. 4), the reducibility at a low temperature range of the catalysts is well associate with the catalytic activity. The Ce-R sample has the best catalytic activity with the largest hydrogen consumption at low temperature. As shown in Supplementary Fig. S5A,B, there is a positive linear relationship between soot oxidation activities (T_10_, T_50_ and T_90_) and the hydrogen consumption of low temperature reduced peak in H_2_-TPR (Fig. 4) under either tight contact or loose contact condition. It is noteworthy that the soot conversion temperatures (T_10_, T_50_ and T_90_) in the presence of NO (Table 3) also present a good linear function with the hydrogen consumption (Supplementary Fig. S6). As the above results, the reduced peak at low temperature is assigned to the reduction of the surface oxygen species on the prepared ceria samples. The more surface oxygen can improve effectively the catalytic activity and it may be an important factor for soot oxidation. XPS results also confirm the above opinions, in which there is also a good linear correction between the surface adsorbed oxygen species and the soot combustion activities of T_10_, T_50_ and T_90_ (Supplementary Fig. S7A,B) under either tight contact or loose contact condition. Thus, the Ce-R sample possesses the maximal amount of surface adsorbed oxygen from H_2_-TPR and XPS results, which leads to its best catalytic activity among these prepared ceria samples. It is really confirmed that the possibility for soot oxidation is related to the surface adsorbed oxygen. Some authors considered that the mobility of lattice oxygen may be a key factor on the soot combustion^46^. However, our results did not show a significant difference in the lattice oxygen mobility of as-prepared ceria. Therefore, in our opinions, the number of surface adsorbed oxygen should play a major role in different activity of soot oxidation over the prepared nanostructure ceria catalysts.

In addition, *in situ* DRIFS results prove that the oxidation capacity of the different nano-structure of ceria by the formation and desorption/decomposition of the surface carbonates species. Carbonate species can decompose completely above 300 °C on the surface of Ce-R. But there are the residual carbonates-like species on the surfaces of Ce-P and Ce-F. In general, the degree of surface carbonate species decomposition is associated with the redox of ceria^52^. The carbonate species on the Ce-R surface occurs to desorption or decomposition at a lower temperature. It means CO_2_ adsorption on the surface of the Ce-R catalyst is weaker than the Ce-P and Ce-F catalysts. Thus, carbonates species is desorbed easily from the Ce-R catalyst surface and exposes a lot of active sites. It is contribute to the cycle of active sites on the catalyst surface. It indicates that the Ce-R surface exist the higher number of surface adsorbed oxygen than the other samples, which is agreement with the results of H_2_-TPR and XPS. Thus, it is further verified that the oxygen species adsorbed on the catalyst surface play crucial role in soot oxidation.

It is noticed that Ce-P exhibits higher activity than the Ce-F for soot combustion in loose contact condition (Table 2 and Fig. 7A); however, the H_2_ consumption of Ce-P is slightly less than that of Ce-F. It indicates that other properties such as surface area may also play an important role in loose contact besides surface adsorbed oxygen. It is found that Ce-P and Ce-R possess a relatively larger S_BET_, which is in the range of 80–88 m^2^/g. As for Ce-F (62 m^2^/g), the surface area is lower than that of the Ce-P and Ce-R catalysts. These results indicate that the high surface area can improve the number of contact points between soot and catalyst^6^, and then enhance the activity in the loose contact. Bueno-López *et al*. also observed an improved activity with the increase in the surface area over CeO_2_ catalyst^54^. Thus, BET surface area may play a role in terms of soot oxidation under the loose contact condition^37^.

In summary, Ce-R sample exhibits the best catalytic activity due to the maximal amount of adsorbed oxygen species on its surface.

## Conclusions

Ceria catalysts with different morphologies including nanorod, nanoparticle and flake have been successfully prepared and their catalytic behaviors were evaluated for soot combustion. CeO_2_ with different morphologies exhibit different physicochemical properties that influence further their catalytic activity of soot combustion. The nanorod CeO_2_ with width of 250 nm and length of about 2 μm exhibited the excellent catalytic activity for soot combustion under loose and tight contact conditions in presence/absence of NO. Its activity is higher than that of some CeO_2_ catalysts that reported in the literatures, and even reached a level of a precious metal catalyst. The characterization results prove that the difference in catalytic activities is attributed to their surface physicochemical properties derived from different morphologies of CeO_2_, in which the adsorbed oxygen species on the surface should be significant factors in soot combustion. In addition, high BET surface area may also be a positive effect on soot oxidation activity under the loose contact conditions.

## Methods

### Catalysts preparation

CeO_2_-nanorod (denoted as Ce-R) has been synthesized through hydrothermal procedure. A typical synthetic procedure is as follow: 0.96 g citric acid and 0.82 g ceria nitrate (Ce(NO_3_)_3_·6H_2_O) were respectively dissolved in 20 and 10 mL distilled water with vigorous magnetic stirring at room temperature. After ten minutes, 1.20 g urea was added into the citric acid solution. Then the ceria nitrate aqueous solution was gradually dropped into the above mixture, and the mixture was stirred for 30 min. The final mixture was transferred to a Teflon-lined autoclave (40 mL) and heated at 120 °C for 24 h. After cooling to room temperature naturally, the fresh precipitates were collected by centrifugation, washed several times with deionized water and ethanol, respectively, and then dried at 80 °C overnight. Ce-R was obtained by calcination of the as-prepared precursor in air at 500 °C for 4 h.

CeO_2_-nanoparticle and CeO_2_-nanoflake (denoted hereafter as Ce-P and Ce-F, respectively) have been synthesized through solvothermal method. Briefly, 1.00 g of Ce(NO_3_)_3_·6H_2_O was dissolved in 30 mL ethanol with vigorous magnetic stirring at room temperature. 1.00 g (to form nanoparticle)/0.50 g (to form nanoflakes) oxalic acid were added into the solution stirred until dissolved, respectively. The mixture was transferred to a Teflon-lined autoclave (40 mL) and heated at 160 °C for 24 h (to form nanoparticle)/12 h (to form nanoflakes). The precipitates were centrifuged and washed with distilled water and ethanol for several times, and dried at 80 °C overnight to obtain the precursor material. Ce-P and Ce-F samples were obtained by calcining the precursors at 500 °C for 4 h.

### Catalysts Characterizations

The power X-ray diffraction (XRD) analysis were measured on a Rigaku D/max-III B diffractometer with a Cu Kα radiation (*λ* = 0.15418 nm), and a working power was 40 kV and 20 mA. SEM images were acquired by using a Hitachi S-4800 microscope at 20 kV. The TEM studies were performed by using a JEM-2010 (JEOL) instrument working with an acceleration voltage of 200 kV. Specific surface area (S_BET_) was measured according to the BET method by nitrogen adsorption at −196 °C using a Tristar 3020 gas adsorption analyzer (Micromeritics). H_2_ temperature-programmed reduction (H_2_-TPR) tests were performed by using TP-5080 equipment. 0.020 g catalyst was pre-treated under 5vol%O_2_/N_2_ stream at 200 °C for 1 h, and then cooled down to room temperature (RT) in the same atmosphere. After purging the sample with 10vol%O_2_/N_2_ for 30 min, the sample was heated to 900 °C at a rate of 10 °C·min^−1^ in 5vol%H_2_/N_2_ (20 mL·min^−1^). H_2_ consumption was recorded using a thermal conductivity detector (TCD), which calibrated with the reduction of known amounts of CuO. X-ray photoelectron spectroscopy (XPS) was recorded using a Kratos-AXIS ULTRA DLD instrument. *In situ* diffuse reflectance Fourier transform IR (DRIFTS) analysis was recorded with a Nicolet 6700 spectrometer. The high temperature diffuse reflectance IR cell was fitted with ZnSe windows. The spectrum resolution was 4 cm^−1^ in the range of 2000–1000 cm^−1^ and 32 scans were collected. Prior to experiment, the sample was pretreated at room temperature for 1 h in a N_2_ stream. Background spectra before CO adsorption were collected in a 40 mL·min^−1^ of N_2_ for 30 min. The sample was exposed in a flowing of 0.5vol%CO/N_2_ (20 mL·min^−1^) until the saturation of the adsorption at room temperature, and then ramped up from 30 to 450 °C in the 0.5vol%CO/N_2_ (20 mL·min^−1^) and 3vol%O_2_/N_2_ (20 mL·min^−1^) mixture streams.

### Catalytic activity measurement

The catalytic activities for soot combustion were evaluated by TG/DTA apparatus (HCP-1 HENVEN Beijing) and measuring the weight loss of catalyst/soot mixtures with the temperature change under different atmospheres: 10vol%O_2_/N_2_ and 1000 ppm NO (when used). Printex-U (Degussa) was used as a model soot. Approximately 9 mg of catalyst were mixed with 1 mg of soot (9:1 on a mass basis), two types of contact conditions were considered, “Loose” contact: the catalyst-soot mixture was prepared by a spatula to homogenize the mixture in a mortar. “Tight” contact: the catalyst-soot mixture was obtained through a pestle to grind soot and catalyst in mortar to reach a close contact. Then, the sample was placed in the sample chamber from 100 to 900 °C at a heating rate of 10 °C·min^−1^ under 10vol%O_2_/N_2_, 1000 ppm NO (when used) with a flow rate of 80 mL·min^−1^ was passed through the reactor. In this work, Temperatures corresponding to 10%, 50% and 90% of weight loss from the TG profile (denoted as T_10_, T_50_ and T_90_, respectively) were taken as indices of the activity for each catalyst. The temperature at which the maximal exothermal value attained in the DTA curve according to the maximal soot combustion rate was denoted as T_m_. The NO_2_ concentration was measured by chemiluminescence NO/NO_X_ Analyzer (CLD 60) provided by ECO Physics AG (Switzerland). The temperature at which the maximal NO_2_ concentration attained in the outlet gas was denoted as T_m_(NO_2_).

## Additional Information

**How to cite this article**: Zhang, W. *et al*. Soot Combustion over Nanostructured Ceria with Different Morphologies. *Sci. Rep.* **6**, 29062; doi: 10.1038/srep29062 (2016).

## Supplementary Material

Supplementary Information

## Figures and Tables

**Table 1:** physicochemical property of Ce-R, Ce-P and Ce-F.

Catalyst	Lattice parameter (Å)	S_BET_ (m^2^·g^−1^)	T_1_^a^ (°C)	T_2_^b^ (°C)	H_T1_^c^ (mmol·g_cat_^−1^)	H_T2_^d^ (mmol·g_cat_^−1^)	O_α1_/O_β_^e^	Ce^3+ ^f (%·atom)
Ce-R	5.4375	80	483	798	0.72	0.45	0.87	25.1
Ce-P	5.4113	88	483	745	0.48	0.43	0.74	16.5
Ce-F	5.4164	62	497	768	0.61	0.39	0.77	19.1

^a^The top temperature of the low-temperature peak.

^b^The top temperature of the high-temperature peak.

^c^The hydrogen consumption at 300–600 °C.

^d^The hydrogen consumption at 700–900 °C.

^e^From O 1s of XPS analysis.

^f^From Ce 3d of XPS analysis.

**Table 2:** Catalytic performances of Ce-R, Ce-P and Ce-F in 10vol%O_2_/N_2_.

Catalyst	Contact condition	T_10_/^o^C	T_50_/^o^C	T_90_/^o^C
Ce-R	Loose	356	500	554
	Tight	286	368	400
Ce-P	Loose	413	521	573
	Tight	320	433	474
Ce-F	Loose	433	554	622
	Tight	306	383	440

**Table 3:** Catalytic performances of Ce-R, Ce-P and Ce-F in 1000 ppm NO and 10vol%O_2_/N_2_ under tight contact conditions.

**Catalyst**	**T**_**m**_**(NO**_**2**_**)/**^**o**^**C**	**T**_**10**_**/**^**o**^**C**	**T**_**50**_**/**^**o**^**C**	**T**_**90**_**/**^**o**^**C**
Ce-R	360	275	315	347
Ce-P	399	301	357	398
Ce-F	379	296	337	372
