# Supplementary material for: Soot Combustion over Nanostructured Ceria with Different Morphologies
Source: Sci Rep. 2016 Jun 29;6:29062. doi: 10.1038/srep29062 (PMC4926248; doi:10.1038/srep29062)
Supplement: Supplementary Information [file srep29062-s1.doc]

**Supplementary Information**

**Soot Combustion over Nanostructured Ceria with Different Morphologies**

Wen Zhang, Xiaoyu Niu, Liqiang Chen, Fulong Yuan*, Yujun Zhu*

Key Laboratory of Functional Inorganic Material Chemistry (Heilongjiang University), Ministry of Education, School of Chemistry and Materials, Heilongjiang University, Harbin, 150080 P. R. China

* Corresponding Author: Yujun Zhu, Email: yujunzhu@hlju.edu.cn, Fulong Yuan, Email: fulongyuan2000@yahoo.com, Tel: +86-451-86609650; Fax: +86-451-86609650

Table S1 Catalytic performance over various morphology pure CeO2 catalysts a

| Preparation method | morphology | T_m_ (^o^C) | Soot model | Soot/catalyst weight | Air condition | References |
| --- | --- | --- | --- | --- | --- | --- |
| Hydrothermal method | nanorods | 368 | Printex-U (Degussa) | 1 mg/9 mg | 10%O_2_/N_2_  80 mL·min^-1^ | This work |
| solvothermal method | nanoparticles | 433 | Printex-U (Degussa) | 1 mg/9 mg | 10%O_2_/N_2_  80 mL·min^-1^ | This work |
| solvothermal method | nanoflakes | 383 | Printex-U (Degussa) | 1 mg/9 mg | 10%O_2_/N_2_  80 mL·min^-1^ | This work |
| Precipitation-hydrothermal | nanocube | 437 | Printex-U (Degussa) | 1 mg/20 mg | O_2_ 60mL·min^-1^ | 37 |
| Precipitation-hydrothermal | nanorod | 425 | Printex-U (Degussa | 1 mg/20 mg | O_2_ 60mL·min^-1^ | 37 |
| Precipitation/ripening | nanofibers | 445 | Printex-U (Degussa) | 5 mg/45 mg | 10%O_2_/N_2_ 100mL·min^-1^ | 38 |
| Hydrothermal method | stars | 410 | Printex-U (Degussa | 5 mg/45 mg | 10%O_2_/N_2_ 100mL·min^-1^ | 38 |
| Precipitation/ripening | fibrous | 375 | Printex-U (Degussa) | 5 mg/45 mg | 10%O_2_/N_2_ 100 mL·min^-1^ | 40 |
| Precipitation/ripening | flakes | 425 | Printex-U (Degussa) | 5 mg/45 mg | 10%O_2_/N_2_ 100 mL·min^-1^ | 40 |
| Precipitation/ripening | sticks | 425 | Printex-U (Degussa) | 5 mg/45 mg | 10%O_2_/N_2_ 100 mL·min^-1^ | 40 |
| Precipitation/ripening | nanofibers | 400 | Printex-U (Degussa) | 5 mg/45 mg | 10%O_2_/N_2_ 100 mL·min^-1^ | 53 |

^a^ All of the catalytic performances were evaluated under “tight contact” conditions.

**References**

37 Aneggi, E., Wiater, D., Leitenburg, C., Llorca. J., A. Trovarelli, Shape-dependent activity of ceria in soot combustion. *ACS Catal.* **4**, 172-181 (2014).

38 Miceli, P., Bensaid, S., Russo, N., Fino, D. CeO_2_-based catalysts with engineered morphologies for soot oxidation to enhance soot-catalyst contact. *Nanoscale. Res. Lett.* **9**, 254 (2014).

40 Kumar, P. A., Tanwar, M. D., Bensaid, S., Russo, N., Fino, D. Soot combustion improvement in diesel particulate filters catalyzed with ceria nanofiber. *Chem. Eng. J.* **207-208**, 258-266 (2012).

53 Bensaid, S., Russo, N., Fino, D. CeO_2_ catalysts with fibrous morphology for soot oxidation: The importance of the soot-catalyst contact conditions. *Catal. Today.* **216**, 57-63 (2013).


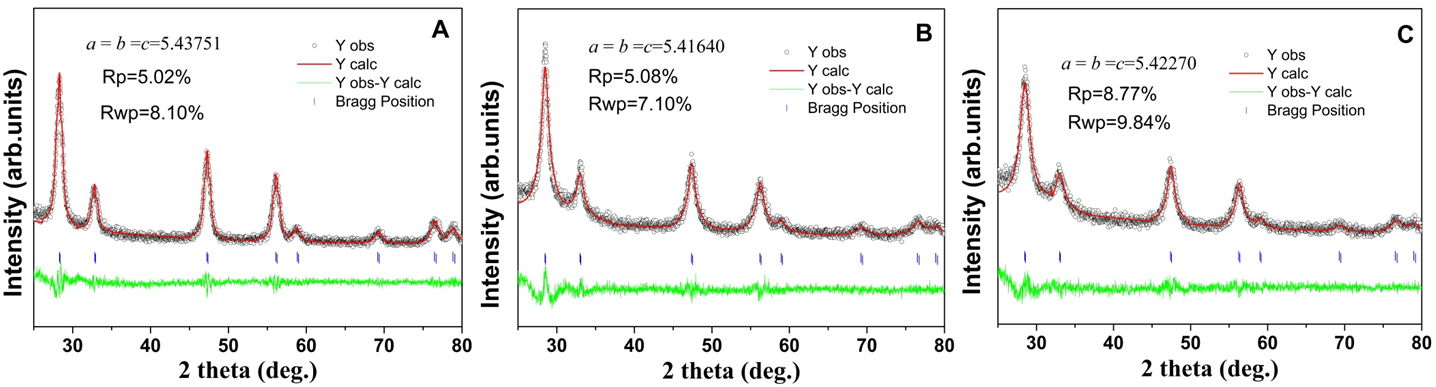


Figure S1 Rietveld analysis profiles of (A) Ce-R, (B) Ce-P and (C) Ce-F


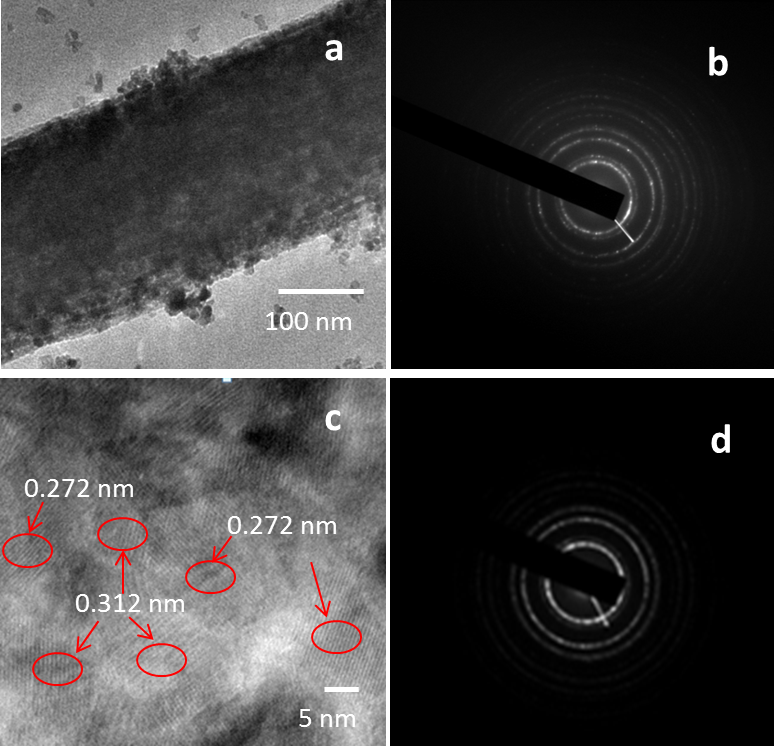


Figure S2 TEM images of (a, b) Ce-R and (c, b) Ce-F

Figure S3 O_2_-TPD curves of (a) Ce-R. (b) Ce-P. (c) Ce-F catalysts


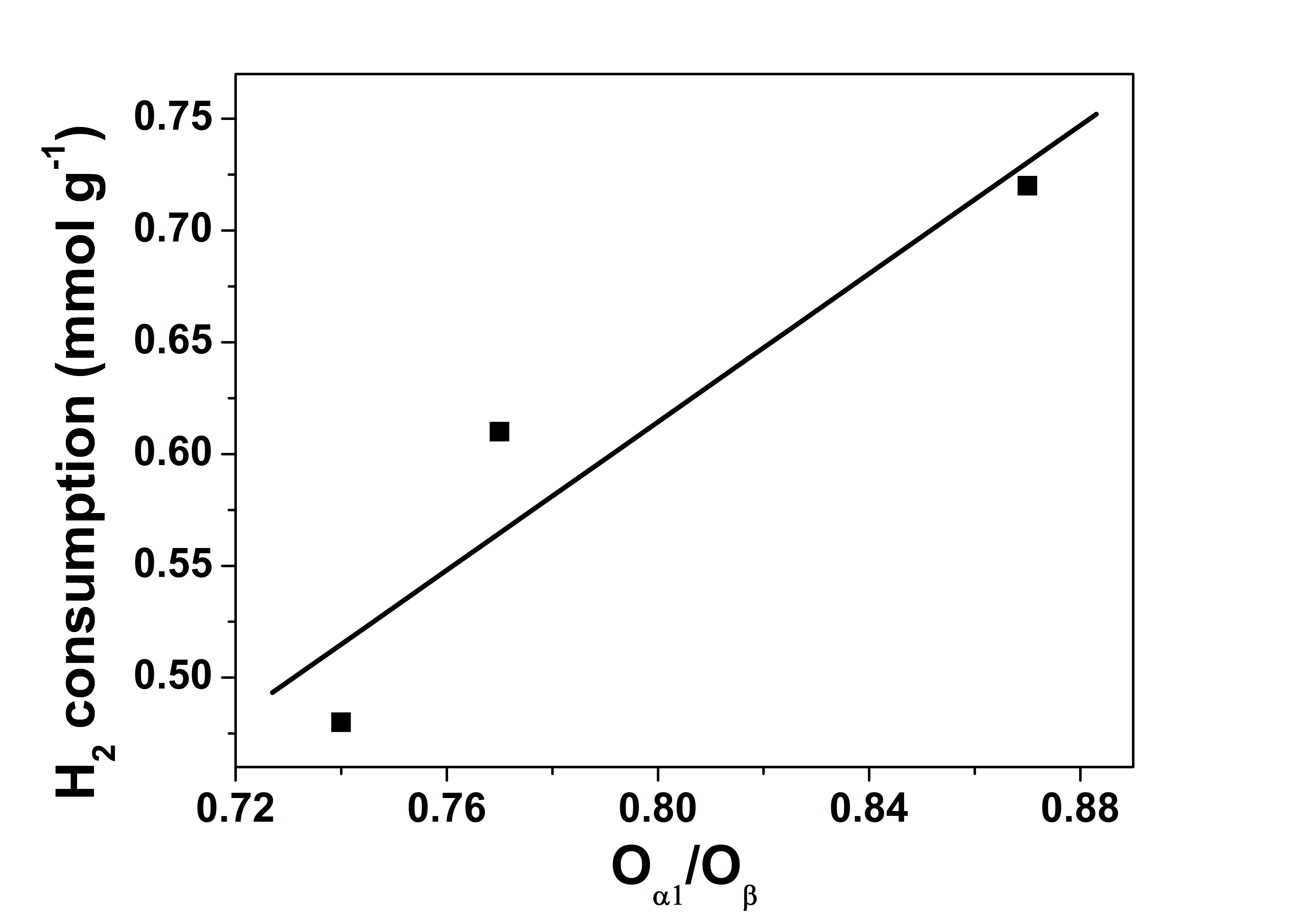


Figure S4 relationship of H_2_ consumption at 300-600 ^o^C and O_α1_/O_β_ from XPS


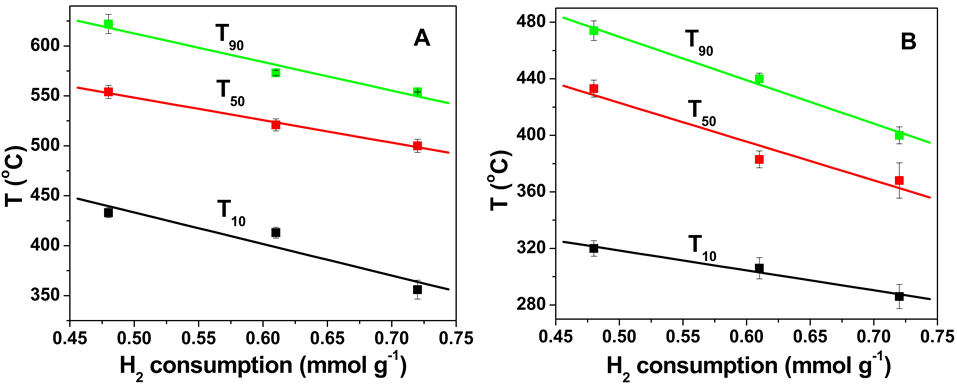


Figure S5 dependence of soot conversion temperatures and H_2_ consumption at 300-600 ^o^C under (A) loose contact (B) tight contact condition in 10vol%O_2_/N_2_


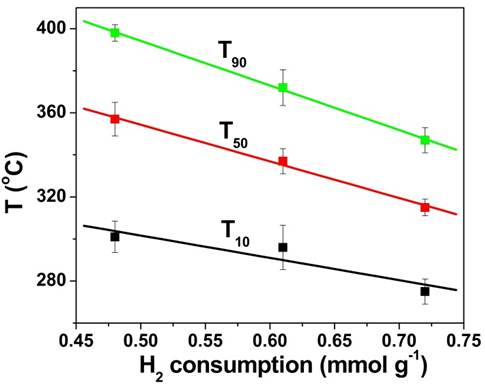


Figure S6 dependence of soot conversion temperatures and H_2_ consumption at 300-600 ^o^C under tight contact condition in 1000 ppm NO and 10vol%O_2_/N_2_


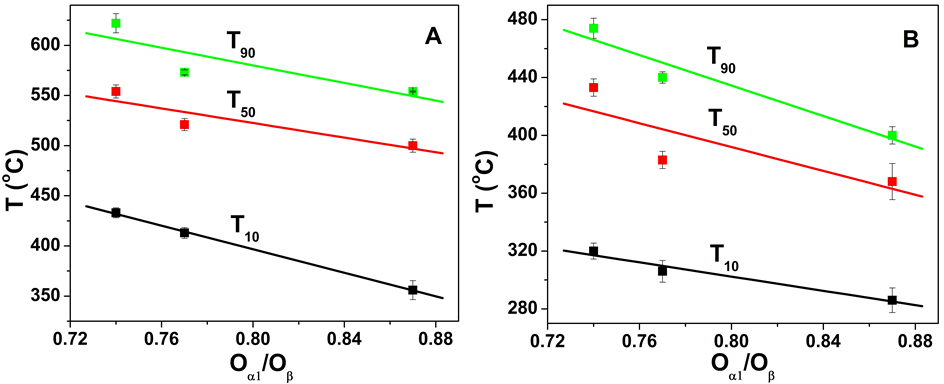


Figure S7 relationship of soot conversion temperatures and O_α1_/O_β_ from XPS under (A) loose contact (B) tight contact condition in 10vol%O_2_/N_2_
